# Cannabidiol inhibits transient receptor potential canonical 4 and modulates excitability of pyramidal neurons in mPFC

**DOI:** 10.3389/fphar.2024.1431758

**Published:** 2024-11-13

**Authors:** Yujun Han, Shuting Wang, Yu Xiang, Liuliu Chang, Xian Wang, Shimin Ren, Fei Guo, Tianyu Li, Zhiqiang Liu, Yang Li

**Affiliations:** ^1^ School of Chinese Materia Medica, Nanjing University of Chinese Medicine, Nanjing, China; ^2^ University of Chinese Academy of Sciences, Beijing, China; ^3^ State Key Laboratory of Drug Research, Shanghai Institute of Materia Medica, Chinese Academy of Sciences, Shanghai, China; ^4^ Shanghai Key Laboratory of Maternal-Fetal Medicine, Department of Anesthesiology, School of Medicine, Shanghai Institute of Maternal-Fetal Medicine and Gynecologic Oncology, Shanghai First Maternity and Infant Hospital, Tongji University, Shanghai, China; ^5^ School of Medicine, Anesthesia and Brain Function Research Institute, Tongji University, Shanghai, China; ^6^ Department of Anesthesiology, Obstetrics and Gynecology Hospital of Fudan University, Shanghai, China; ^7^ National Clinical Research Center for Aging and Medicine, Huashan Hospital, Fudan University, Shanghai, China

**Keywords:** TRPC4, cannabidiol, epinephrine, inhibitor, medial prefrontal cortex

## Abstract

Cannabidiol (CBD), a non-psychoactive compound derived from the cannabis plant, has been extensively studied for its potential therapeutic effects on various central nervous system (CNS) disorders, including epilepsy, chronic pain, Parkinson’s disease, and stress-related neuropsychiatric disorders. However, the pharmacological mechanisms of CBD have not been fully elucidated due to the complexity of their targets. In this study, we reported that the transient receptor potential canonical 4 (TRPC4) channel, a calcium-permeable, non-selective cation channel, could be inhibited by CBD. TRPC4 is highly abundant in the central nervous system and plays a critical role in regulating axonal regeneration, neurotransmitter release, and neuronal network activity. Here, we used whole-cell electrophysiology and intracellular calcium measurements to identify the inhibitory effects of CBD on TRPC4, in which CBD was found to inhibit TRPC4 channel with an IC_50_ value of 1.52 μM TRPC4 channels function as receptor-operated channels (ROC) and could be activated by epinephrine (EP) via G proteins. We show that CBD can inhibit EP-evoked TRPC4 current *in vitro* and EP-evoked neuronal excitability in the medial prefrontal cortex (mPFC). These results are consistent with the action of TRPC4-specific inhibitor Pico145, suggesting that TRPC4 works as a functional ionotropic receptor of CBD. This study identified TRPC4 as a novel target for CBD in the CNS and suggested that CBD could reduce the pyramidal neuron excitability by inhibiting TRPC4-containing channels in the mPFC.

## Introduction

Cannabidiol (CBD), a prominent cannabinoid found in cannabis, has little affinity for classical cannabinoid receptors (CB1 and CB2) with fewer reported side effects than Δ9-THC (Landucci et al., 2022). CBD is known for its non-psychoactive nature and possesses numerous beneficial pharmacological properties, such as anti-inflammatory and antioxidant effects (Atalay et al., 2019; Gurgenci et al., 2023). Currently, accumulating evidence suggests that CBD has a regulatory effect on CNS disorders, such as antipsychotic and anxiolytic properties (Blessing et al., 2015; Bonn-Miller et al., 2021; Soares and Campos, 2017; Spinella et al., 2021; Szkudlarek et al., 2021). For instance, the administration of CBD, whether systemically or injected into specific brain areas, has been shown to reduce anxiety-related behaviors in rodents caused by previous exposure to acute restraint stress (Campos and Guimarães, 2008; Fogaça et al., 2014). As for the clinical field, a randomized study demonstrated that CBD reduces positive psychotic symptoms in schizophrenia adults (McGuire et al., 2018).

CBD is now believed to interact with various molecular targets. It has been shown that many phytocannabinoids and endocannabinoids can modulate Transient Receptor Potential (TRP) channels (Muller et al., 2018). Some researchers also categorize TRP channels as “ionotropic cannabinoid receptors”. To date, three TRP subfamilies have been identified that can be modulated by cannabinoids: TRPV, TRPM, and TRPA. CBD has been found to have activating effects on TRPV1, TRPV2, TRPV3, TRPA1, and antagonistic effects on TRPM8 (De Petrocellis et al., 2011; De Petrocellis et al., 2012; Qin et al., 2008). However, there is currently a lack of experiments demonstrating the interaction between CBD and the TRPC family.

Transient receptor potential canonical 4 (TRPC4) is a non-selective cation channel that is widely distributed in multiple brain regions. TRPC4 is considered a receptor-operated channel (ROC), which can be regulated by G protein-coupled receptors (GPCRs) (Lee et al., 2005). Most studies have shown that the activation of G proteins is required for the TRPC4 activation, until englerin A (EA), a TRPC4/TRPC5 direct agonist was discovered and used in research. For instance, activation of M2 receptors via G_i/o_ proteins plays a direct role in TRPC4 opening (Zholos et al., 2024). Histamine could trigger a transient TRPC4-induced current by histamine H1 receptor (Obukhov and Nowycky, 2002). The activation of TRPC4 allows Ca^2+^ and monovalent cations to inflow into the cytoplasm, leading to cell depolarization and increased intracellular Ca^2+^ concentration (Chen et al., 2020; Philipp et al., 1998), which is crucial for neuronal excitability. A range of studies have systematically explored the association between TRPC4 and CNS disorders, for example, the activation of TRPC4 led to anxiety, fear-related responses, and major depressive disorder (Nazıroğlu and Demirdaş, 2015). In contrast, TRPC4 inhibitors, such as HC-070 and M084, have been reported to significantly alleviate depressive and anxiety-related behaviors (Just et al., 2018; Yang et al., 2015). In this study, we investigated the inhibitory effect of CBD on TRPC4 and examined its modulatory action on pyramidal neurons of the mPFC.

The mPFC represents a highly evolved brain region that is relevant to cognitive functions, emotion regulation, and social stress response. Stress can lead to an imbalance of the excitation-inhibition ratio. For example, in an electrophysiological study, the stress model mice exhibited increased excitability in mPFC neurons (Yang et al., 2016). Therefore, the excitability of mPFC neurons may be an important indicator of stress-related states.

Epinephrine (EP), a catecholamine primarily found in the peripheral nervous system, could also influence synaptic transmission and neuronal activity in the brain. It has been confirmed that some nervous system responses to stress are usually positively correlated with the concentration of EP, such as anxiety, PTSD, and panic (Martinho et al., 2020; Southwick et al., 1999; Theron et al., 2023; van Zijderveld et al., 1999). Canonically, epinephrine and norepinephrine act via adrenergic receptors, a family of GPCRs. Adrenergic receptors can be divided into two groups, α and β, with 9 subtypes in total. As mentioned above, TRPC4 is considered a receptor-operated channel and is often regulated by GPCR. A previous study reported that TRPC4 could be a sensor for catecholamine neurotransmitters such as epinephrine and dopamine via G proteins in enterochromaffin cells (Bellono et al., 2017). We verified that EP could activate TRPC4 via adrenergic receptors in heterologous expression system, and CBD could inhibit EP-evoked TRPC4 activity. Furthermore, we observed the effect of EP on the excitability of medial prefrontal cortex (mPFC) neurons, which may depend on the TRPC4-containing channel.

In this study, we identified TRPC4 as a novel target of CBD by using electrophysiological recordings and intracellular calcium measurements. CBD was able to inhibit EA- or EP-evoked TRPC4 current *in vitro*, and could effectively attenuate the neuronal excitability triggered by EP in mPFC. These insights expand the targets of CBD and shed light on the potential neural and molecular mechanisms through which CBD exerts its pharmacological effects in CNS.

## Results

### Inhibition effect of CBD on TRPC4 channel

**FIGURE 1:**
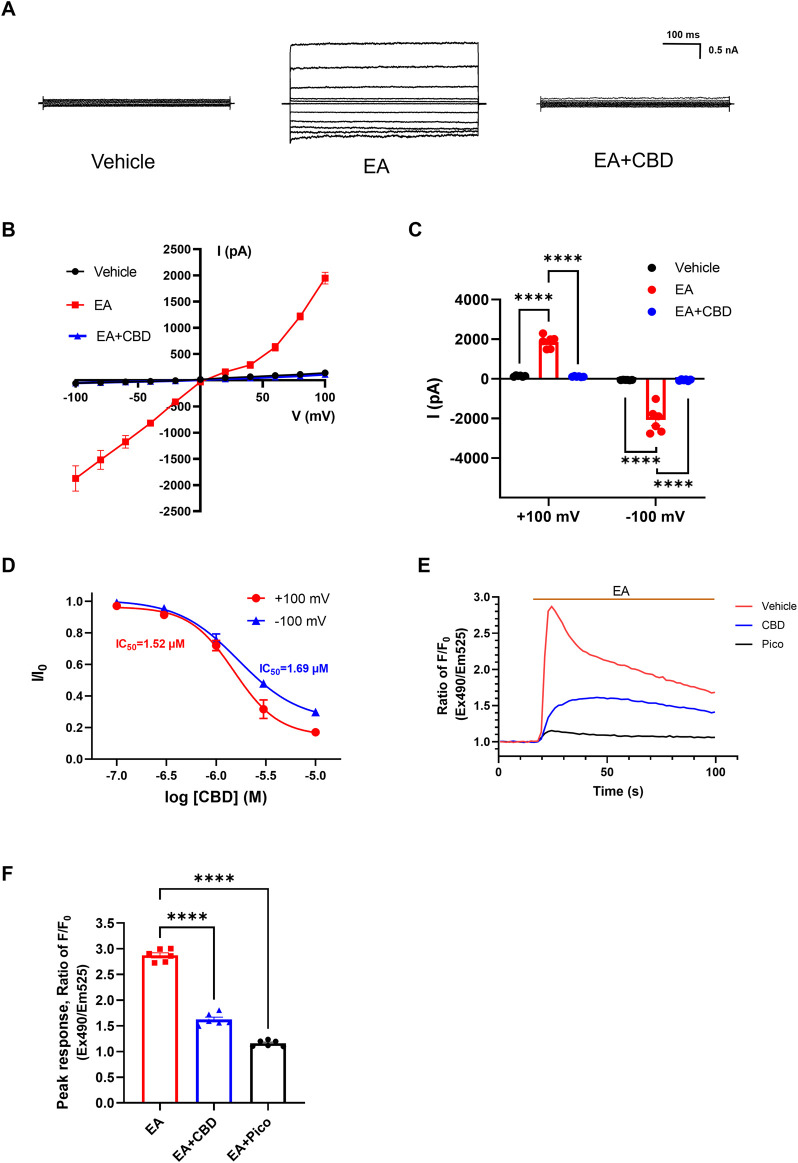
Inhibition effect of CBD on TRPC4 channels. **(A)** Presentative whole-cell current traces of HEK293-TRPC4 cells from −100 to +100 mV within vehicle bath solution (left), 10 nM EA administration (middle), 10 nM EA + 30 μM CBD (right). **(B)** The whole-cell current-voltage relation of TRPC4 over-expressed in HEK293 cells, n = 8. **(C)** The statistics of TRPC4 whole-cell average current value in **(B)**. Two-way-ANOVA followed by Tukey test was used for statistical analysis. **(D)** Dose-dependent inhibition of TRPC4 by CBD, n = 3 in each concentration. **(E)** Intracellular Ca^2+^ trace in CHO–TRPC4 cells response to EA (75 nM) among three different treated groups: vehicle HBSS solution (red), preincubated with 1 μM Pico145 (black), preincubated with 50 μM CBD (blue), n = 6. **(F)** Normalized peak value from each group in **(E)**, n = 6. Brown-Forsythe (F [2.000, 6.312] = 126.5, *p* < 0.0001) and Welch (W [2.000, 7.255] = 126.5, *p* < 0.0001) ANOVA followed by Dunnett test was used for statistical analysis. The data are shown as the mean ± s. e.m., *** indicates *p* < 0.001.**** indicates *p* < 0.0001.

Given the extensive research on cannabinoids targeting TRPV, TRPM, and TRPA members, we sought to investigate whether cannabinoids also regulate TRPC channels. Here, we used patch-clamp electrophysiology and intracellular calcium measurements to explore the CBD effect for TRPC4. Firstly, we performed whole-cell patch clamp recordings in HEK293 cells transfected with TRPC4. EA is a widely recognized potent agonist on TRPC4. As expected, 10 nM EA robustly activated the TRPC4 current. The outward current at +100 mV increased from 140.3 ± 11.82 to 1865 ± 128.4 pA, and the inward current at −100 mV increased from −58.58 ± 3.129 to −2081.0 ± 269.5 pA (Figures 1A–C). The treatment of CBD caused a strong inhibition of the EA-evoked current both outward and inward, with the peak current decreasing to 113.7 ± 8.640 pA (outward current) and −60.27 ± 15.04 pA (inward current) (Figures 1A–C). In the CHO cells, CBD could inhibit EA-evoked TRPC4 current as well (Supplementary Figures S1A, B). Subsequently, we explored the dose-response relationship of CBD on TRPC4. The constructed concentration-response curve revealed a dose-dependent manner of CBD on the TRPC4 channel, with a calculated half-maximal inhibition concentration (IC_50_) of 1.52 ± 0.6 μM (outward current) and 1.68 ± 0.6 μM (inward current) (Figure 1D).

As a non-selective cation channel, activation of TRPC4 increased Ca^2+^ permeability and facilitated the Ca^2+^ influx, a phenomenon associated with increased cellular excitability (Han, Trinidad and Shi, 2015). Considering that, then we performed intracellular calcium measurements to evaluate the function of TRPC4 and the effects of CBD on TRPC4. Applying 75 nM EA significantly increased the Ca^2+^ influx of HEK293-TRPC4 cells with a peak ratio of about 2.87 ± 0.05 fold before and after EA administration. However, EA failed to elicit an increased Ca^2+^ signal in cells preincubated with 1 μM Pico145 (a TRPC4 inhibitor) (Rubaiy et al., 2017), with the peak ratio about 1.16 ± 0.02 fold before and after EA administration. Similarly, cells preincubated with 50 μM CBD exhibited a significantly reduced response in intracellular Ca^2+^ concentration to EA compared to cells treated with EA alone, with a peak ratio of about 1.62 ± 0.05 fold before and after EA administration (Figures 1E, F). A similar fluorescence trend was seen in CHO cells (Supplementary Figures S2A, B). Additionally, EA lost its effect in two conditions: depleting extracellular calcium (with 10 mM calcium chelator EGTA) and using non-transfected cells, suggesting that the fluorescent signal is exclusively induced by TRPC4-mediated Ca^2+^ influx (Supplementary Figures S2C, D). In summary, Our results strongly suggest that CBD inhibits the TRPC4.

### CBD inhibits TRPC4 current evoked by EP

**FIGURE 2:**
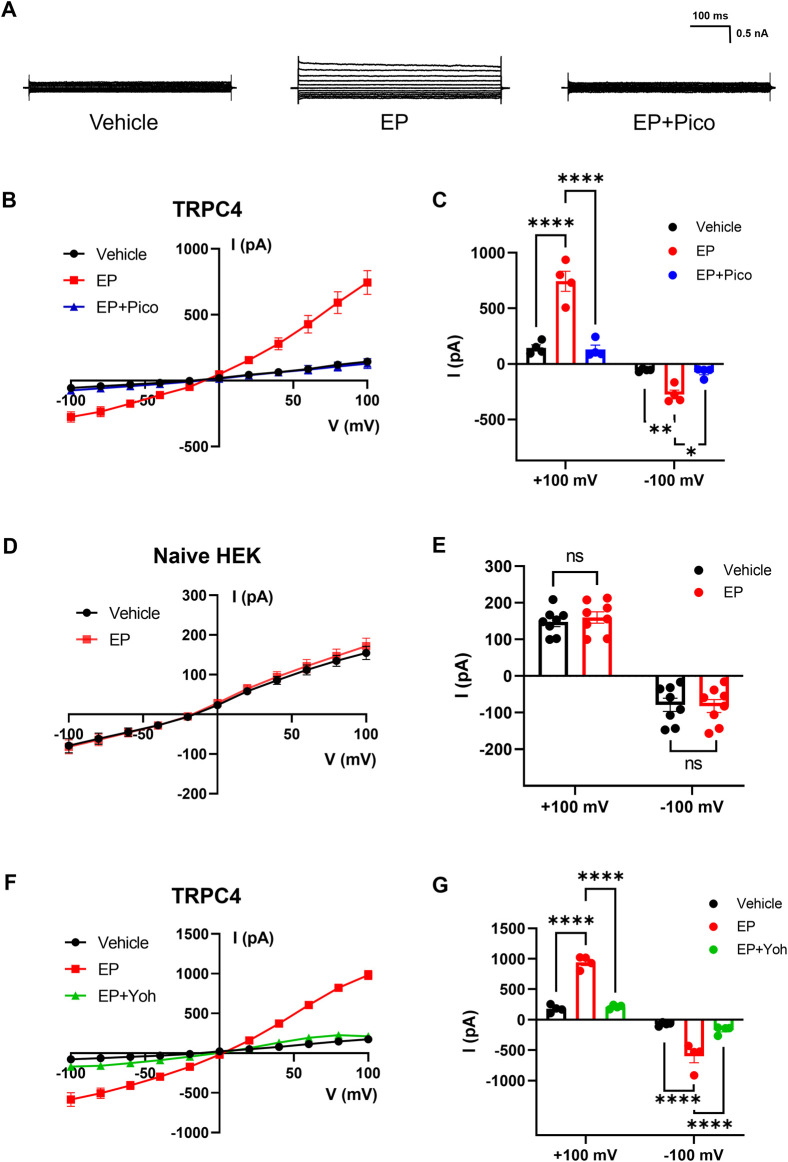
Activation effect of EP on TRPC4. **(A)** Presentative whole-cell current traces recorded of HEK293-TRPC4 cells from −100 mV to +100 mV within bath solution (left), 30 μM EP (middle), 30 μM EP + 1 μM Pico145 (right). **(B)** The whole-cell current-voltage relation of TRPC4 activated by 30 μM EP and inhibited by 1 μM Pico145. **(C)** The statistics of whole-cell average current value in **(B)**, n = 4. Two-way-ANOVA followed by Tukey test was used for statistical analysis. **(D)** The whole-cell current-voltage relation recording from untransfected HEK293 cells under bath solution or 30 μM EP application. **(E)** The whole-cell average current value in **(D)**, n = 8. Two-way-ANOVA followed by Tukey test was used for statistical analysis. **(F)** The whole-cell current-voltage relation of TRPC4 activated by 30 μM EP and inhibited by 30 μM yohimbine. **(G)** The statistics of whole-cell average current value in **(F)**, n = 4. Two-way-ANOVA followed by Tukey test was used for statistical analysis. The data are shown as the mean ± s. e.m., * indicates *p* < 0.05, ** indicates *p* < 0.01, *** indicates *p* < 0.001, **** indicates *p* < 0.0001 ns indicates no significance.

TRPC4 is widely accepted as receptor-operated cation channels, which can be activated by G protein-coupled receptors through the downstream signaling pathways. For example, it has been suggested that TRPC4 could respond to neurotransmitters targeting G protein-coupled receptors via G_i/o_ and G_q/11_ pathways, including acetylcholine, dopamine, epinephrine, and glutamate (Bellono et al., 2017; Myeong et al., 2018). In this study, we performed whole-cell voltage-clamp recording on HEK293 cells overexpressed TRPC4 to verify the effect of EP on TRPC4. As shown in Figure 2A, middle panel, the application of 30 μM EP to the bath solution led to a substantial increase in the current, with the outward current rising from 143.8 ± 28.06 to 743.9 ± 90.06 pA and the inward current rising from −56.08 ± 6.613 to −275.4 ± 38.67 pA. The application of 1 μM Pico145 revealed a pronounced inhibition of both outward and inward current, with peak current decrease to 129.9 ± 38.25 pA (outward current) and −74.93 ± 22.41 pA (inward current) (Figures 2A–C). Non-transfected cells were also used to record the whole-cell current response to EP. The whole-cell current was not significantly altered by EP application (Figures 2D, E), indicating that the background whole-cell current of HEK293 cells was not affected by EP. These results suggest that the current activated by EP before is specifically mediated by TRPC4. Next, we want to verify whether endogenous adrenergic receptors are involved in the EP activation process to TRPC4. We used pharmacological approach to manipulate related receptors and assess their impact on the activation of TRPC4. Yohimbine is an α1 and α2 receptor antagonist. The application of 30 μM yohimbine significantly decreased the activation current induced by EP (Figures 2F, G), which suggested that the activation of EP on TRPC4 depends on adrenergic receptors.

**FIGURE 3:**
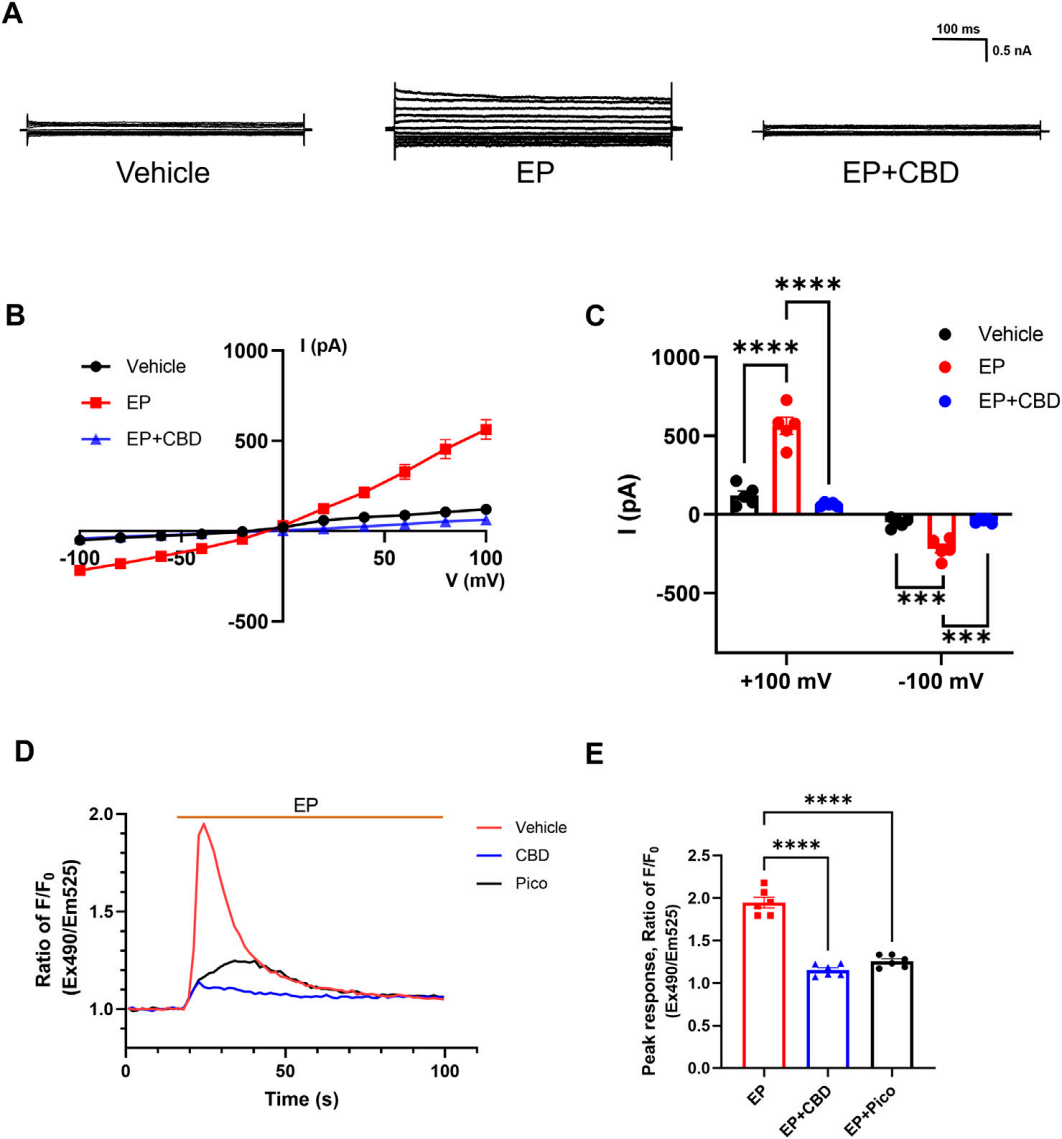
CBD inhibits EP-evoked TRPC4 activation. **(A)** Presentative whole-cell current traces recorded of HEK293-TRPC4 from −100 mV to +100 mV within bath solution (left), 30 μM EP (middle), 30 μM EP + 30 μM CBD (right). **(B)** The whole-cell current-voltage relation of TRPC4 activated by 30 μM EP and inhibited by 10 μM CBD. **(C)** The statistics of whole-cell average current value in **(B)**, n = 5. Two-way-ANOVA followed by Tukey test was used for statistical analysis. **(D)** Intracellular Ca^2+^ trace over time in HEK293-TRPC4 cells responses to EP (50 μM) among three different treated groups: vehicle HBSS solution (red), preincubated with 1 μM Pico145 (black), preincubated with 50 μM CBD (blue), n = 6. **(E)** Normalized peak value from each group in **(D)**. One-way-ANOVA (F [2, 15] = 139.8, *p* < 0.0001) followed by Tukey test for statistical analysis. The data are shown as the mean ± s. e.m., *** indicates *p* < 0.001, **** indicates *p* < 0.0001.

Based on the activation effect of EP on TRPC4, we further investigate the influence of CBD on TRPC4 current evoked by EP. The TRPC4 current was evoked by EP administration, with the outward current changing from 120.2 ± 28.48 to 563.1 ± 53.57 pA and the inward current changing from −50.86 ± 13.68 to 218.3 ± 28.57 pA, followed by a rapid decline to 63.42 ± 5.293 pA (outward current) and −41.37 ± 6.248 pA (inward current) after the administration of 10 μM CBD (Figures 3A–C). It indicates that CBD can inhibit EP-evoked TRPC4 current. A similar electrophysiology trend was also seen in CHO cells (Supplementary Figures S1C, D). Furthermore, EP could also significantly increase the Ca^2+^ influx of HEK293-TRPC4 cells with a peak ratio of about 1.95 ± 0.06 fold before and after EP administration. The application of 50 μM CBD and 1 μM Pico145 almost abolished EP response with peak ratio of about 1.15 ± 0.03 and 1.26 ± 0.03 fold, respectively, before and after EP administration (Figures 3D, E). A similar fluorescence trend was seen in CHO cells (Supplementary Figures S2E, F). Depletion of extracellular calcium by EGTA blocked the EP-induced Ca^2+^ signal, proving that the process was driven by extracellular Ca^2+^ influx (Supplementary Figure S2G). EP also lost its effect in non-transfected cells, suggesting that the fluorescent signal is exclusively induced by TRPC4-mediated Ca^2+^ influx (Supplementary Figure S2H). These results indicate that CBD can inhibit TRPC4 activated by EP signaling, suggesting that phytocannabinoids may regulate GPCR-triggered receptor-operated Ca^2+^ entry through TRPC4.

### CBD modulates neuron excitability induced by EP via TRPC4 in mPFC

Previous studies have demonstrated that TRPC4 contributes to the Ca^2+^-permeable responses to GPCR signaling in a variety of cells, including the gut epithelium enterochromaffin cells, adrenal chromaffin cells, neurons, smooth muscle cells, and others (Bellono et al., 2017). Catecholamines are an important class of neurotransmitters in the CNS. Considering that the majority of catecholamine receptors are GPCRs, we hypothesized that TRPC4 could respond to catecholamines such as EP through the adrenergic receptor signaling in the CNS. In this study, we performed whole-cell patch-clamp recordings on mPFC pyramidal neurons in brain slices to explore whether TRPC4 is also involved in neuronal response to EP signaling and whether the inhibitory effect of CBD on TRPC4 affects this process.

**FIGURE 4:**
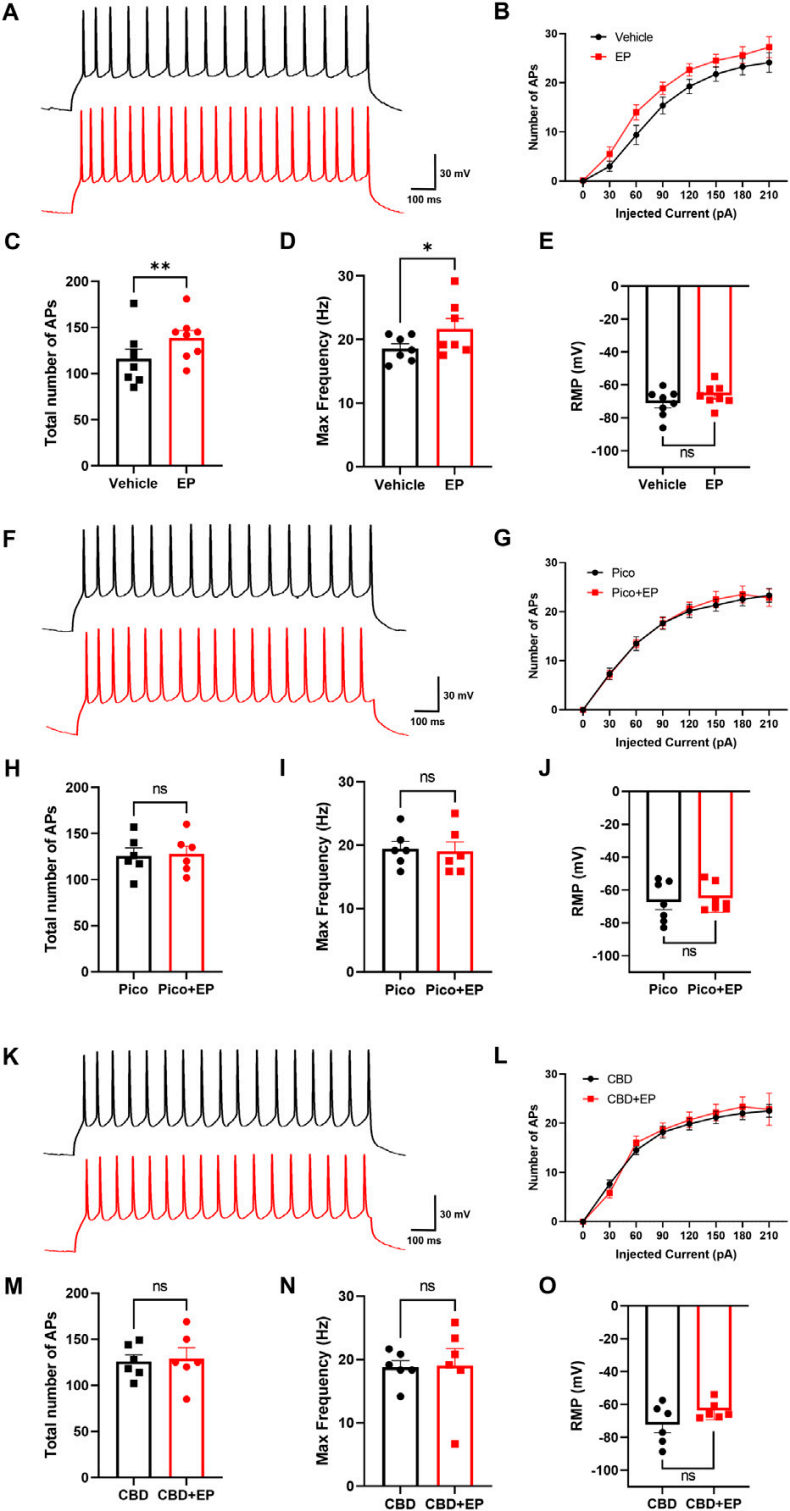
Modulation effect of EP and CBD in mPFC pyramidal neurons. **(A)** Representative trace of APs at +90 pA from mPFC neurons in the baseline (black), and 30 μM EP application (red). **(B)** The statistics of AP firing number under each injected current value from 0 to 210 pA. **(C–E)** The statistics of the total number of APs **(C)**, max firing frequency **(D)**, membrane potential **(E)** of recorded neurons, n = 8 cells from 3 mice. **(F)** Representative trace of APs at +90 pA from mPFC neurons preincubated with 10 μM Pico145 in the baseline (black), and 30 μM EP application (red). **(G)** The statistics of AP firing number under each injected current value from 0 to 210 pA. **(H–J)** The statistics of the total number of APs **(H)**, max firing frequency **(I)**, the membrane potential **(J)** of recorded neurons, n = 7 cells from 3 mice **(K)** Representative trace of APs at +90 pA from mPFC neurons preincubated with 10 μM CBD in the baseline (black), and 30 μM EP application (red) **(L)** The statistics of AP firing number under each injected current value from 0 to 210 pA **(M–O)** The statistics of the total number of APs **(M)**, max firing frequency **(N)**, the membrane potential **(O)** of recorded neurons, n = 6 cells from 3 mice. The data are shown as the mean ± s. e.m., paired *t*-test was used for statistical analysis, * indicates *p* < 0.05, ** indicates *p* < 0.01, ns indicates no significance.

Both TRPC4 and adrenergic receptors are widely expressed in mPFC (Cash et al., 1986; Castelli et al., 2016; Fowler et al., 2007; Pillay et al., 2020; Riccio et al., 2002), which is an important region that regulates multiple behaviors and emotions. The PrL layer V was chosen as the recorded region due to its abundant distribution of pyramidal neurons, identified based on their classical neuronal form (Supplementary Figure S3A). In order to measure the effect of agents on neuronal excitability, we recorded action potential through the current injection of 0–210 pA. We first examined the effect of EP on the mPFC pyramidal neurons. Upon perfusion of 30 μM EP into the recording chamber, the excitability of pyramidal cells rapidly increased, with the total APs number of pyramidal neurons sharply increasing from 116.1 ± 10.24 to 138.5 ± 8.31 and the max frequency of AP increasing from 20.10 ± 1.67 s^−1^ to 22.71 ± 1.75 s^−1^ (Figures 4A–D). However, the resting membrane potential (RMP) of the neurons did not change significantly (Figure 4E). These phenomena align with the neuronal response to TRPC4 activation, including the increase of firing rate due to the influx of Ca^2+^ and Na^+^ (Fowler et al., 2007; Uzay and Kavalali, 2022; Wang et al., 2007). Given that, to investigate the role of TRPC4 in EP-enhanced excitability, we preincubated the brain slices with the TRPC4 antagonist Pico145 in the recording chamber for 30 min. Result data indicated that by suppressing TRPC4, EP had no effect in terms of the total APs number, max frequency of AP, and RMP (Figures 4F–J). The results suggest that the excitatory effect of EP is TRPC4-dependent.

Next, based on the inhibitory effect of CBD on TRPC4 in the heterologous expression system, we investigated whether CBD could block neuronal excitability triggered by EP. After the brain slices were preincubated with 30 μM CBD for 30 min, the addition of EP could not significantly change the neuronal excitability and RMP (Figures 4K–O). This data shows that CBD can block the effect of EP on pyramidal neuron excitability in mPFC, which is similar to the role of TRPC4 inhibitor Pico145. Additionally, there was no significant difference in neuron excitability and membrane potential when brain slices were incubated with Pico145, CBD, or vehicle ACSF before EP was administrated (Supplementary Figures S3B–D), indicating that the function of Pico145 and CBD appears to be focused on modulating the EP-response process in the recorded neurons instead of the constitutive neuronal excitability. In summary, we observed that EP could directly enhance the excitability of pyramidal neurons in mPFC, which could be blocked by suppressing TRPC4 using Pico145 and CBD.

## Discussion

It is widely agreed that TRP channel is an important kind of cannabinoid target outside of CB1 and CB2. Both phytocannabinoids and endocannabinoids significantly intersect with ligands of TRP channels (Muller et al., 2018). Six members of TRPA, TRPV and TRPM family have been found that could be modulated by cannabinoids (Muller and Reggio, 2020). However, there are few reports on the effects of cannabinoids on TRPC family. In this study, we reported that CBD, the major non-psychoactive component of cannabis, has an inhibitory effect on TRPC4. CBD can inhibit both small-molecule agonist (EA) and GPCR-induced TRPC4 current, which shows TRPC4 is a potential cannabinoid target.

TRPC4 is a calcium-entry nonselective cation channel. It has been reported that some agents could bind and block TRPC4-containing channels. The apo structure of mouse TRPC4 was resolved in 2018 (Duan et al., 2018; Vinayagam et al., 2018). Recently, the structure of TRPC4 in complex with three small molecule inhibitors, GFB-8438, GFB-9289, and GFB-8749 (Vinayagam et al., 2020). All these three pyridazinone-based inhibitors bind in the cavity form by voltage-sensor-like domain (VSLD) and TRP helix, which is in the intracellular side of the transmembrane region and next to the S1-S4 helices (Vinayagam et al., 2020). Based on the molecular docking within this structure we have done, we speculate that CBD resides in this cavity as well. Our future work will focus on identifying the binding site of CBD and verifying the residues of TRPC4 interacting with CBD by loss-of-function mutations. Besides, this cavity is not consistent with either the cannabidiol site or the cannabinoid non-cannabidiol site on TRPV2 (Pumroy et al., 2019; Su et al., 2023; Zhang et al., 2022), which may be one of the structural bases for the opposite effects of cannabinoids on TRPC4 and TRPV2.

TRPC4 and TRPC5 have a close relationship and share many biophysical characteristics. Both of them could be activated by EA and potentiated by G-protein-coupled receptors (Wang et al., 2020). We will study the effects of CBD on other TRPC members in future work, such as TRPC5 and TRPC1. TRPC4 is capable of coassembling with TRPC5 and TRPC1 to form heteromeric channels in a variety of tissue/cell types (Goel et al., 2002; Hofmann et al., 2002). Therefore, exploring the effects of cannabinoids on different heteromultimers is helpful to better explore the regulation of cannabinoids on TRPC4-containing channels in physiological processes.

The inhibitors of TRPC4/5 have the potential for the treatment of CNS diseases and renal diseases (Wang et al., 2020). Pico145 (also known as HC-608), a xanthine-based TRPC4/5 inhibitor, is widely used in pharmacology studies because of its high potency. This study also uses it as a control compound (Rubaiy et al., 2017). However, the poor physiochemical properties limit its use *in vivo*. Despite its numerous potential targets, CBD, as well as other cannabinoids, is still valuable in the research of diseases targeting TRPC4/5 because of its good useability and strong potency.

It is generally considered that the activation of the TRPC4 channel requires the activation of G proteins unless using small-molecule agonists. Activation of GPCR triggers both receptor- and store-operated Ca^2+^ influx of TRPC4. In this study, we used EP to activate adrenergic receptors to mimic the GPCR regulation of TRPC4. It was reported that TRPC4 contributes to the Ca^2+^-influx downstream of adrenoreceptor activation in enterochromaffin cells (Bellono et al., 2017). We also determined EP could actively heterologously express TRPC4 via adrenergic receptors. CBD strongly inhibits EP-induced TRPC4 responses, suggesting that phytocannabinoids may regulate the catecholamine system *in vivo*.

TRPC4 was found to be abundant in the corticolimbic regions of the brain and was implicated in fear and anxiety-like behaviors. Behavioral studies with *Trpc4* ^*−/−*^ mice showed remarkable anxiolytic and antidepressant action (Riccio et al., 2014). This anxiolytic effect could be replicated by HC-070, a TRPC1/TRPC4/TRPC5 pan-inhibitor (Just et al., 2018). These results imply the therapeutic potential of TRPC4-containing channel inhibitors to treat anxiety disorders. In addition, the anxiety-relieving effects of CBD have been extensively researched in both human and animal studies (Crippa et al., 2018; Guimarães et al., 1990; Moreira et al., 2006). We focus on mPFC, a brain region highly associated with emotion, to perform functional studies of TRPC4 *ex vivo*. The excessive excitability of PFC pyramidal neurons related to the anxious state (Kuang et al., 2022), for instance, increased excitement in layer V pyramidal neurons of the mPFC was found in anxiety-like behaviors mice (Kabir et al., 2017; Li et al., 2021). We performed brain slice clamp-patch recording in mPFC and observed the regulated function of CBD to EP-induced neuron excitement, which is consistent with the effect of Pico145, the TRPC4 inhibitor. This result suggests CBD may reduce EP-induced neuronal overexcitation by inhibiting TRPC4 in the mPFC. However, more precise target validation needs to be done in the future, and behavioral tests are necessary to verify whether CBD exerts its anxiolytic effects through TRPC4.

Some studies have described the presence and function of EP in the CNS (Gupta et al., 1984; Rayevsky et al., 2012; Wang et al., 2016), although the conventional perspective suggests that EP primarily operates within the peripheral system, while norepinephrine (NE) is the dominant player in the CNS. Additionally, it should be noted that NE can be converted into EP through the action of the enzyme PNMT (Tank and Lee Wong, 2015). In this experiment, we directly observed the strong regulatory effect of EP on neuronal excitability, which seems to mimic the response of mPFC to stress. Nevertheless, it remains unclear how the concentration of EP fluctuates and influences neuronal activity in mPFC. We found that CBD could inhibit the effects of EP in the mPFC, suggesting that TRPC4 may be a common target of both. This provides a new insight to explain the pharmacological effects of CBD and EP. For example, excess release of EP could exacerbate fear memory (Alves et al., 2016; Oliveira et al., 2018), while CBD and endocannabinoids have been reported to modulate fear extinction (Gunduz-Cinar et al., 2023). In addition, TRPC4-containing channel may also be involved in other processes regulated by CBD in the CNS, such as epilepsy (von Wrede et al., 2021; Zanelati et al., 2010), social stress (Brancato et al., 2023), and mood disorders (Linge et al., 2016; Zanelati et al., 2010).

In summary, our study revealed a potential new target for CBD to act on the CNS. It highlights a potential connection among cannabinoids, neurotransmitters, and TRPC channels in neuronal regulation, offering a novel view of the pharmacological mechanisms of CBD at the molecular and neuronal levels. We will further explore the molecular mechanism of CBD target on TRPC4 and look forward to more studies on the regulation of cannabinoids on TRPC channels in the future.

## Materials and methods

### Animals

Male C57BL/6J mice aged 3–4 weeks were used for brain slice experiments. Mice were raised under stable conditions with access to food and water *ad libitum*. All animal care and experimental procedures complied with the National Institutes of Health Guide for the Care and Use of Laboratory Animals and approved by the Institute Animal Care and Use Committee at Shanghai Institute of Materia Medica, Chinese Academy of Sciences.

### Chemicals

Englerin A (Cat # HY-133168) and Pico145 (Cat # HY-101507) were purchased from MedChem Express. Cannabidiol was kindly provided by Kunming University of Science and Technology. Yohimbine HCl (Cat # S2373) was purchased from Selleck Chemicals (±)-Epinephrine hydrochloride (Cat #E4642) was purchased from Sigma-Aldrich.

### Molecular biology and cell transfection

Human embryonic kidney 293 (HEK293) cells (ATCC) were cultured in DMEM containing 4.5 g/L glucose and L-glutamine. Chinese hamster ovary (CHO) cells were provided by the Cell Bank of the Chinese Academy of Sciences (Shanghai, China). CHO cells were maintained in DMEM/F12. All medium was supplemented with 10% fetal bovine serum (FBS, Gibco) and 100 μg/mL penicillin-streptomycin. The cells were cultured at 37°C in a humidified atmosphere with 5% CO_2_. The human TRPC4a channel was cloned into the pIRES2-EGFP vector between BamHI and XhoI sites (Genewiz). Cells were plated in 35 mm tissue culture dishes and transfected with the 1 μg plasmids per dish using Lipofectamine 3000 (Thermo Fisher Scientific) following the manufacturer’s protocol.

### TRPC4-expressed HEK293 or CHO cell electrophysiology

All electrophysiological recordings were obtained using patch-clamp recordings for macroscopic current dose−response curves. All patch-clamp recordings were conducted 24–48 h after transfection. Patch-clamp recordings were done with an Axopatch-200B amplifier and Axon Digidata 1550 A driven by Clampex10 software (Molecular Devices). The microelectrodes fashioned from 1.5 mm thin-walled borosilicate glass with filament were pulled from a Flaming/Brown type micropipette puller (P-97, SUTTER INSTRUMENT) with the resistances of 3–7 MΩ for whole-cell recordings. The bath solutions contained the following: 135 mM NaCl and 10 mM HEPES, 10 mM Glu, 5 mM KCl, 2 mM CaCl_2_, 1 mM MgCl_2_, pH 7.4. The pipette solution contained the following: 140 mM KCl, 10 mM HEPES, 0.5 mM EGTA, 3 mM Mg-ATP, and pH 7.3. All recordings were performed at room temperature (approximately 25°C). The current signals were filtered at 1 kHz and digitized at a 10 kHz sampling frequency. Whole-cell currents were evoked by 500 m voltage steps from −100 mV to +100 mV in 20-mV increments, followed by stepping down to 0 mV. The access resistance of each patch is greater than 800 MΩ. The series resistance was compensated before forming a tight patch seal. Liquid junction potentials were less than 2 mV calculated by JPCalc software. The drug reached its final concentration by perfusion within 10s using ALA-VM8 (Scientific Instruments).

### Intracellular calcium measurements

After 24-hours transfection, cells were seeded at a density of 80,000 cells per well in 96-well plates and, the next day were incubated in 3 μM Cal-520 AM-HBSS (Hanks balanced salt solution, 0.5% Bovine Serum Albumin, 250 μM Sulfinpyrazone) for 90 min at 37°C. After that, the dye-containing solution was replaced, and test compounds were administered for 10 min at room temperature. Cells were stimulated with englerin A or epinephrine solution, and fluorescence was recorded every 1.6 s (excitation 490 nM/emission 525 nM) for 100 s using Flexstation 3 (Molecular Device, United States). Baseline fluorescence (F_0_) is the average fluorescence value obtained during 17 s before adding the agonist. The normalized fluorescence ratio was acquired by dividing the fluorescence value by the average baseline fluorescence (RFU F/F_0_).

### Brain slice electrophysiology

Brain slices containing the mPFC were prepared from male mice (21 days old). Mice were anesthetized with sodium pentobarbital (40 mg/kg, i. p.) and sacrificed by decapitation. The brains were quickly removed and placed in cold (4°C) oxygenated (95% O_2_ and 5% CO_2_) artificial cerebrospinal fluid (ACSF) containing 124 mM NaCl, 2.5 mM KCl, 2 mM CaCl_2_,1 mM MgSO_4_, 25 mM NaHCO_3_, 1 mM NaH_2_PO_4_ and 10 mM glucose. For containing coronal brain slices (300 μM), the brains were glued to the cutting staged tissue slicer (Leica, VT1000S). The cutting solution and ACSF were constantly bubbled with a mixture of 95% oxygen and 5% carbon dioxide. Slices were transferred to a submerged recovery chamber with oxygenated (95% O_2_ and 5% CO_2_) ACSF at room temperature for at least 1 h. The chamber was continuously perfused with ACSF or drug solutions at a rate of 4–5 mL/min, and its temperature was maintained at 33°C ± 1°C. The pipette solution contained the following: 3 mM Mg-ATP, 120 mM KCL, 10 mM EGTA, 1 mM MgCl_2_, 10 mM HEPES, pH 7.2 with KOH. AP firing output was calculated as the number of APs that responded to 1200-ms depolarizing current injections (0–210 pA, steps of 30 pA).

## Statistics

Whole-cell patch-clamp data were collected and analyzed with Clampex 10.2 and Clampfit 10.2 (Molecular Devices). Whole-cell voltage clamp experimental data of the inhibition of TRPC channels by CBD were fitted using the dose−response function. The half-inhibition concentrations of CBD were derived from fits of the dose−response curves to the function:

$$\frac{I}{I_{0}}=A_{1}+\frac{A_{2}-A_{1}}{1+10^{\left(\log x_{0}-x\right)p}}$$

where I_0_ and I are current amplitudes before and after the application of inhibitors; A1 and A2 are constants between 0 and 1; x is the concentration of the inhibitor; x_0_ is the concentration when 50% inhibition was reached (IC50); and *P* is the Hill constant. The data in the calcium measurements experiment, represented as N, indicates replicate numbers in columns of the 96-well plate.

Statistical analyses, including two-tailed Student's t-test, one-way ANOVA, Brown-Forsythe, and Welch ANOVA, were conducted using GraphPad Prism 9.0. In statistical analyses, the experimental data were expressed as the mean ± standard error of the mean (s.e.m.). Experiments were independently repeated at least three times with similar results. *p* values below 0.05 were considered statistically significant. The statistical details of experiments can be found in the results and figure legends.

## Data Availability

The original contributions presented in the study are included in the article/Supplementary Material, further inquiries can be directed to the corresponding authors.

## Supplementary material

Supplementary Figure 1 CBD inhibits TRPC4 current in the CHO cells. **(A)** The whole-cell current-voltage relation of TRPC4 over-expressed in CHO cells, n = 6. **(B)** The statistics of TRPC4 whole-cell current value at +100 mV in (a). Repeated measures one-way ANOVA with Geisser-Greenhouse correction (F [1.075, 5.375] = 813.1, *p* < 0.0001) followed by Tukey test was used for statistical analysis. **(C)** The whole-cell current-voltage relation of TRPC4 activated by 30 μM EP and inhibited by 10 nM CBD. **(D)** The statistics of whole-cell current value at +100 mV in (d). Repeated measures one-way ANOVA with Geisser-Greenhouse correction (F [1.309, 4.158] = 74.23, *p* = 0.0008) followed by Tukey test was used for statistical analysis. The data are shown as the mean ± s.e.m., ** indicates *p* < 0.01, **** indicates *p* < 0.0001.

Supplementary Figure 2 CBD inhibits TRPC4-mediated Ca^2+^ influx in the CHO cells. **(A)** Intracellular Ca^2+^ trace in CHO-TRPC4 cells response to EA (75 nM) among three different treated groups: vehicle HBSS solution (red), preincubated with 1 μM Pico145 (black), preincubated with 50 μM CBD (blue), n = 6. **(B)** Normalized peak value from each group in (a). One-way-ANOVA (F [2, 15] = 453.3, *p* < 0.0001) followed by Tukey test for statistical analysis. **(C)** Intracellular Ca^2+^ trace response to EA (75 nM) of three treated groups in extracellular Ca^2+^ free buffer, n = 6. **(D)** Intracellular Ca^2+^ trace of non-transfected CHO cells and CHO-TRPC4 cells responded to EA (75 nM). **(E)** Intracellular Ca^2+^ trace in CHO-TRPC4 cells responses to EP (50 μM) among three groups, n = 6. **(F)** Normalized peak value from each group in (e). One-way-ANOVA (F [2, 15] = 102.2, *p* < 0.0001) followed by Tukey test for statistical analysis. **(G)** Intracellular Ca^2+^ trace response to EP (50 μM) of three treated groups in extracellular Ca^2+^ free buffer, n = 6. **(H)** Intracellular Ca^2+^ trace of non-transfected CHO cells and CHO-TRPC4 cells responded to EP (50 μM). The data are shown as the mean ± s.e.m., **** indicates *p* < 0.0001.

Supplementary Figure 3 CBD incubation did not affect action potential frequency and membrane potential of pyramidal neurons in mPFC. **(A)** Representational picture of recorded pyramidal neuron morphology from mPFC layer V. **(B-D)** The statistics of max firing frequency (b), total number of APs (c), and the membrane potential (d) of recorded pyramidal neurons in different incubation (ACSF/Pico145/CBD) before EP application, all data origin from Figure 4. The data are shown as the mean ± s.e.m., one-way ANOVA followed by Tukey test was used for statistical analysis. F [2, 16] = 0.2123, p = 0.8103 in (b); F [2, 17] = 0.3943, *p* = 0.6802 in (c); F [2, 18] = 0.3959, *p* = 0.6788 in (d). ns indicates no significance.
