# Supplementary figures and images for: Cannabidiol inhibits transient receptor potential canonical 4 and modulates excitability of pyramidal neurons in mPFC
Source: Front Pharmacol. 2024 Nov 13;15:1431758. doi: 10.3389/fphar.2024.1431758 (PMC11603362; doi:10.3389/fphar.2024.1431758)

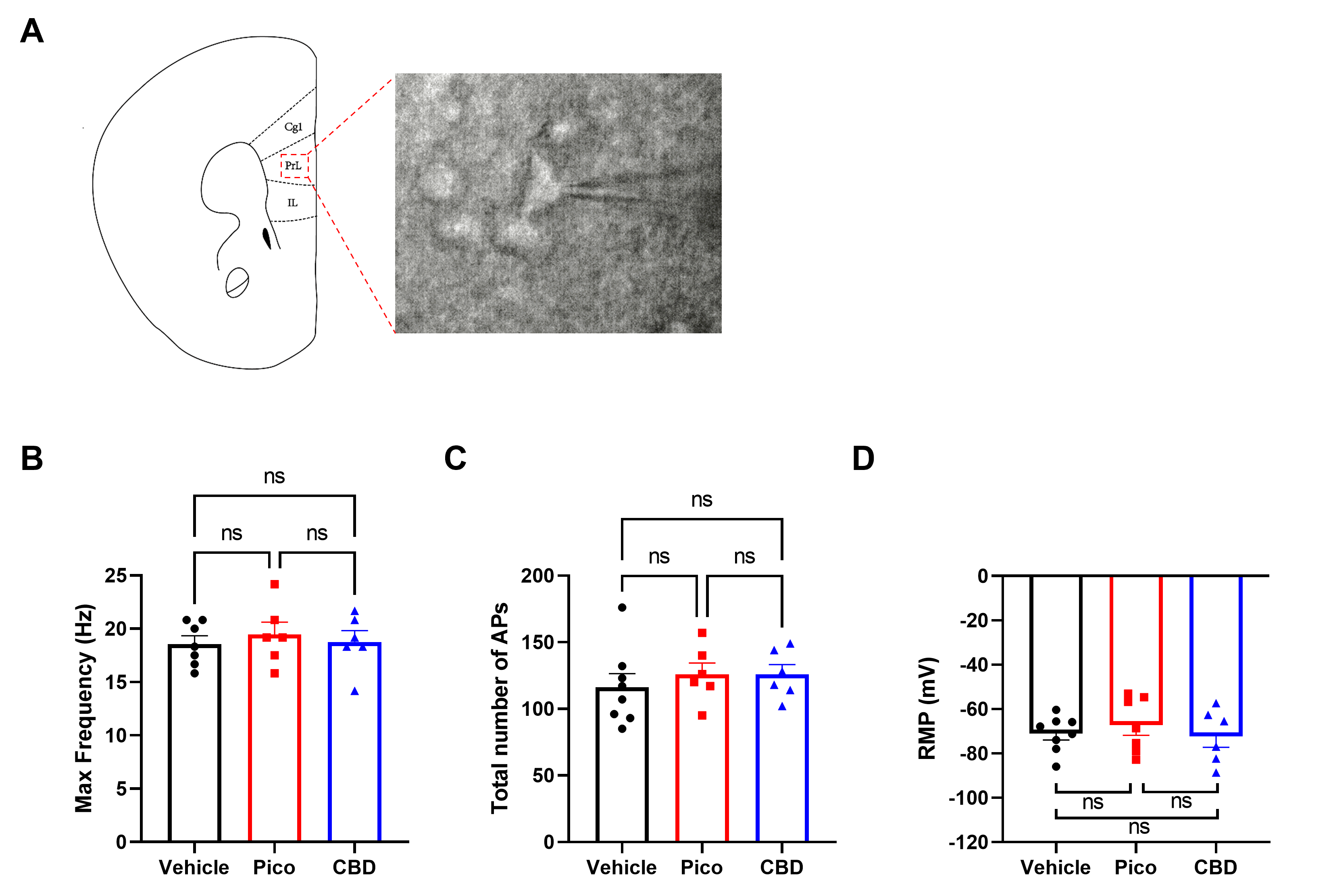

Supplement: Supplementary file 1 [file Image3.tif]

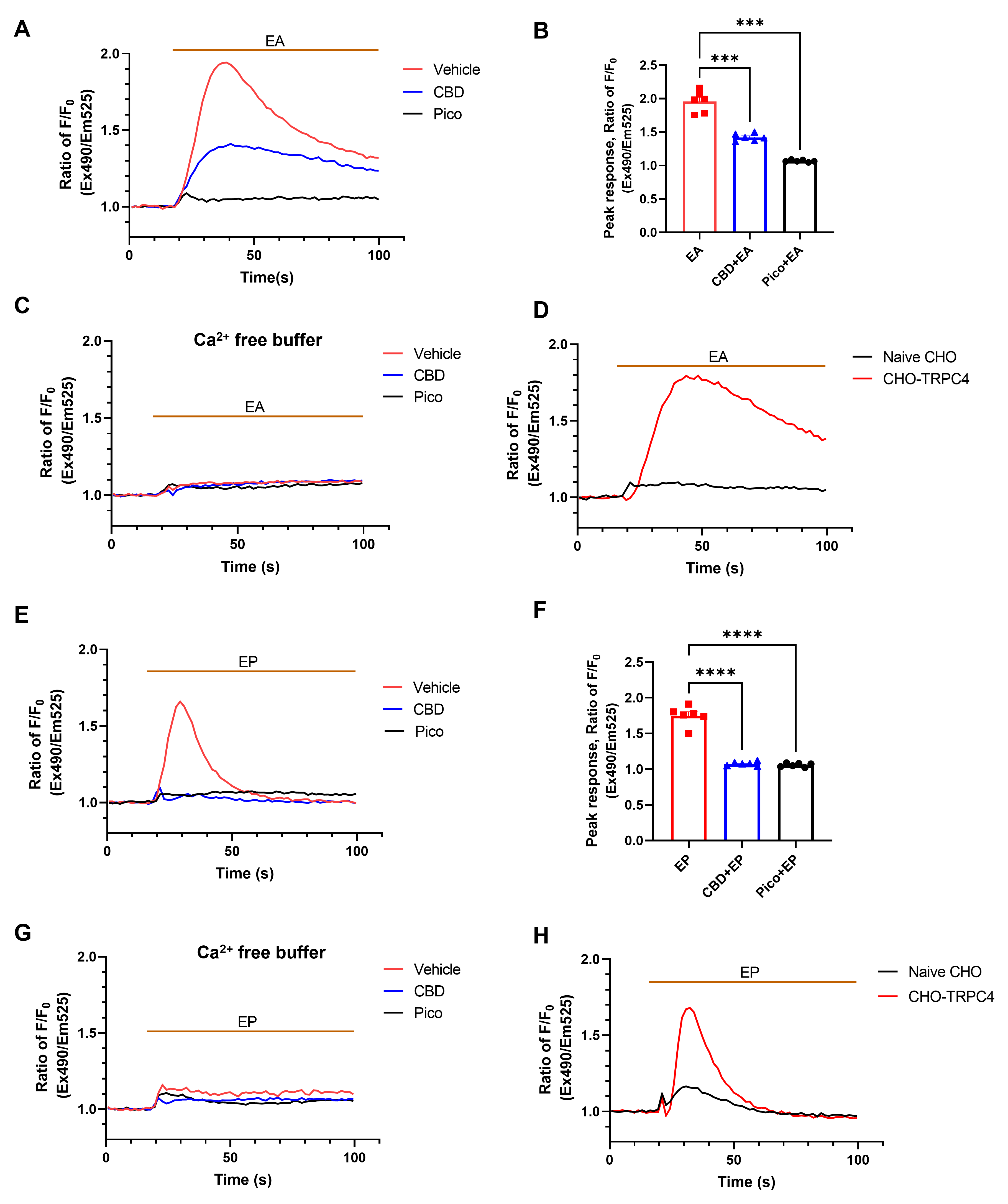

Supplement: Supplementary file 2 [file Image2.tif]

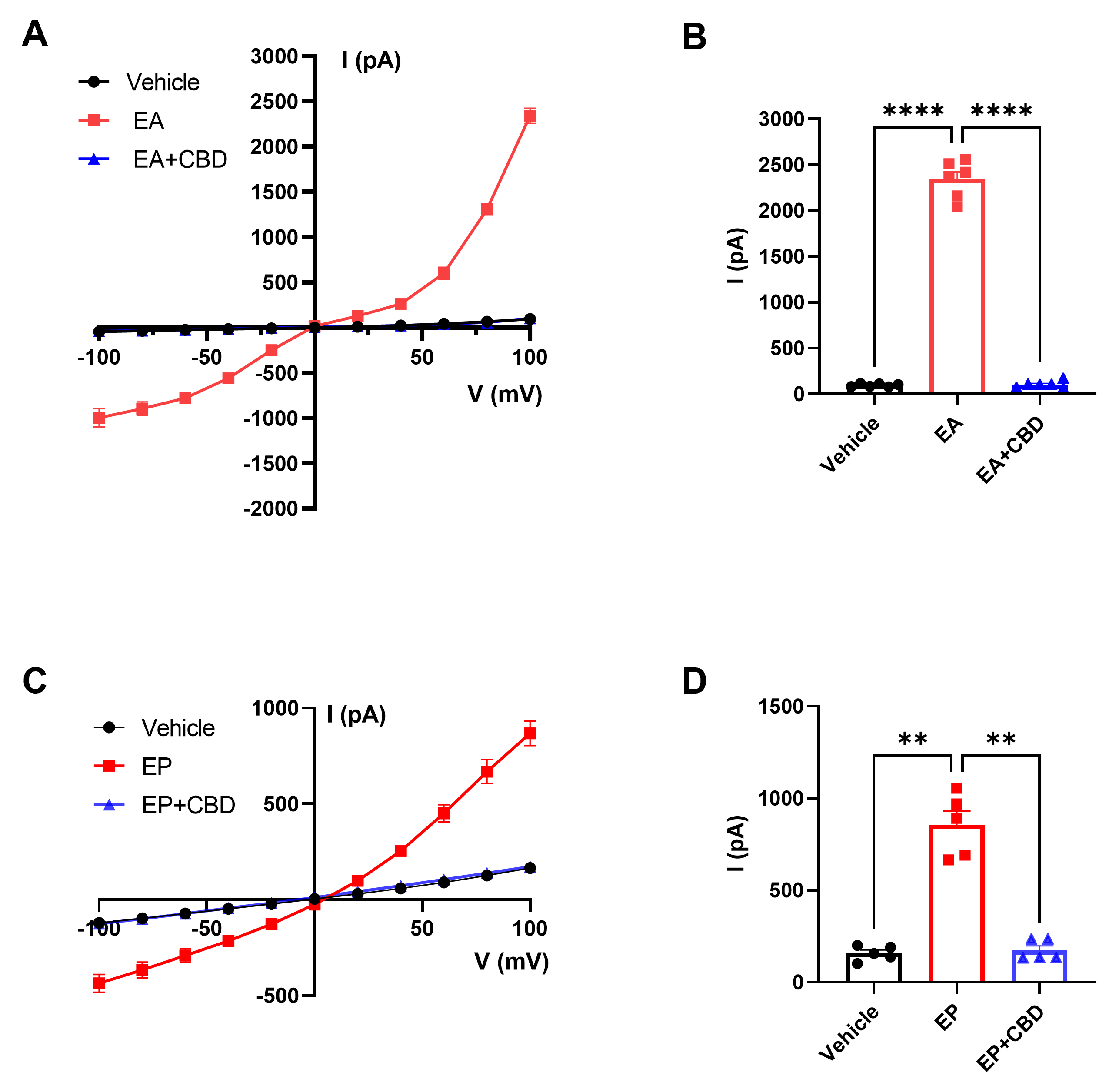

Supplement: Supplementary file 3 [file Image1.tif]
